# Observations and Reflections on the Sympathies Existing between the Stomach and Intestines with the Brain, and on Some of the Diseases of Those Organs, Depending on the Predominance of Their Vital Powers in the System during Infancy

**Published:** 1820-08

**Authors:** Richard Harrison

**Affiliations:** Fellow of the Royal College of Physicians, &c.


FOR THE LONDON MEDICAL AND PHYSICAL JOURNAL.

*Observations and Reflections on the Sympathies existing between the Stomach and Intestines with the Brain, and on some of the Diseases of those Organs, depending on the Predominance of their Vital Powers in the System during Infancy.* Read before the Royal College of Physicians of London, in the year 1820, as a Gulstonian Lecture, by Richard Harrison, M.D. F.L.S. Fellow of the Royal College of Physicians, &c.

THE functions of the stomach and brain are so intimately connected, that a disorder of the one generally occasions a disturbance of the other; and their respective sympathies are frequently so immediate, as to baffle even the most experienced practitioner and most acute observer, in his attempt to discover which is the primary, and which the secondary, seat of various disorders.

A severe blow on the head not only occasions disturbance of the stomach, but even green or black bile will be frequently vomited in consequence of it; and, when a person first goes to sea, he is in general attacked with nausea and vomiting, in consequence of the disturbance of the brain, appearing as vertigo, &c. which he then suffers. In these familiar examples, there can be no doubt but that the stomach is only sympathising with the diseased condition of the brain.

On the other hand, when the stomach has been loaded with an excess of food, or such as is indigestible, we not unfrequently find, as a consequence, vertigo, impaired vision, and great confusion of the internal senses, and other signs of disturbance of the functions of the brain. In these examples, also, we are not at a loss to determine the seat of the disease.

But there are many instances in which it is difficult to ascertain which is the primary, and which the secondary, affection; and yet it is of the first importance that this obscurity should be removed, that we may be enabled to resort to the appropriate remedies.

When we reflect on the offices in the animal economy performed by the stomach, so essential to the existence of life, that we find some imperfect animals without any evident traces of heart, circulation, or brain, the stomach alone being sufficient to maintain their general vitality, it cannot escape the most superficial observer, that diseases of this viscus must spread their baneful effects to the functions of the other organs; and we readily perceive that this is accomplished, to a great extent, through the intervention of the brain.

Aliments or medicines received into the stomach sooner or later influence every part of the body, through the medium of the brain and nervous sytem: for, to what other power can we ascribe the sudden strength and vigour diffused over the languid, debilitated invalid, by the taking of grateful food or stimulant medicines. It was the consideration of these facts that induced Van Helmont and his disciples to place the seat of the soul at its cardiac orifice.

The brain, also, on being excited to vigorous action, imparts more powerful nervous energy to the stomach, and produces a more perfect and quicker digestion.

Thus hope and joy first act on the brain, and, by its sympathising influence on the stomach, the whole body becomes strengthened, and the gastric fluid is copiously secreted, and digestion facilitated. Pleasurable impressions will even enable a person to support considerable fatigue for a long time with little food.

On the other hand, the depressing passions debilitate the cerebral functions, and hence induce a correspondent disorder of the stomach. These effects are frequently very immediate and evident. Thus, sorrowful events communicated to a person in perfect health before dinner, will destroy the most craving appetite.

This circumstance did not escape the observation of Shakspeare. Henry the Eighth, on presenting Cardinal Wolsey with his intercepted letter to the Pope, is made to say,

> "Read over this;
> And after, this; and then to breakfast, with
> What appetite you may."

Having made these general remarks on the intimate connection subsisting between the brain and stomach, let us now notice their effects in some particular diseases.

Mechanical injuries of the head furnish us with a clear and marked example of the influence which the brain exerts over the stomach. These injuries occasion severe disorders of the last-mentioned organ and of the intestines. The tongue is furred, there is indigestion, and the bowels are obstinately constipated; and these symptoms severely torment the patient even for days after the cerebral disturbance has been removed.

Sometimes these injuries occasion the disorder to assume the character of idiopathic dyspepsia.

Although apoplexy depends immediately on lesion of the functions of the brain, it very rarely occurs without an accompanying diseased condition of the stomach; and in some cases the vomiting is so severe, that to a superficial observer it would appear to be only a disease of the stomach.

Indigestible food in the alimentary canal has frequently induced, by its sympathetic irritation, an attack of apoplexy. This has led many practitioners to the empirical practice of exhibiting powerful emetics on all occasions.

This pernicious practice demands our attention. Vomiting is in a great measure accomplished by the contractions of the voluntary muscles, and these derive their nervous influence from the brain. If the brain be severely oppressed, or disorganized, it can no longer transmit its nervous influence to those muscles; hence vomiting does not take place.

With a view to the practical deductions we shall draw from the influence the injuries of the head have over the stomach, we may remark that, when the injury, a fracture and depression of a portion of the cranium for instance, is in the degree only which disturbs, but does not destroy, the functions of the brain, nausea and vomiting occur; but, when compression has considerably disturbed the functions of the brain, no signs of disordered stomach take place; but immediately the depressed bone is removed, then the brain being relieved, nausea and vomiting come on.

Vomiting may, I think, be thus explained: the irritation of the stomach makes a call on the brain for the aid of the diaphragm and the abdominal muscles, in order to expel its contents. The diaphragm then becomes contracted and fixed, the ribs drawn down, and the abdominal muscles drawn inwards; so that the stomach is pressed on all sides by voluntary muscles, which, with its own contraction, expel its contents. This union of action is generally so intimate in the adult, that vomiting never takes place without it.

Those who, in opposition to Magendie, have stated that it is the contraction of the stomach alone which causes vomiting, have not taken a sufficiently comprehensive view of that act; and Magendie, seeing the importance of the action of the muscles, has attributed too much to their agency, in the results of his experiments on this subject.

The first experiment consisted in giving a dog six grains of tartar-emetic: as soon as nausea was excited, an incision was made in the linea alba, in the direction of the stomach; the finger was then introduced into the cavity of the abdomen, to ascertain whether the stomach underwent any contraction. On each nausea taking place, the finger was strongly compressed from above, by the liver being depressed by the diaphragm, and from below, by the intestines being compressed by the abdominal muscles. The stomach also seemed to be compressed; but, instead of contracting, seemed on the contrary to increase in volume. The nausea was then more frequently repeated; and, on vomiting taking place, the finger was very strongly compressed. The stomach evacuated in part its contents, but was itself in no respects sensibly contracted. On the nausea intermitting for some moments, the opening was enlarged, in order to bring the stomach into sight; which being accomplished, it made forcible efforts to escape by the enlarged incision; but this was prevented by the hand of the operator. The nausea recurred in a few minutes, and the stomach was seen, to the surprise of the experimenter, to become filled with air, and increased to three times its size: vomiting soon followed this distension; and it was obvious to all the by-standers, that the stomach was compressed without its muscular fibres undergoing the least contraction. The organ voided the air and a portion of the aliments; but, immediately after these had been discharged, it remained flaccid, and only after some minutes gradually contracted upon itself, and returned to nearly the same dimensions as it occupied before the vomiting took place. This experiment was repeated, and constantly afforded the same results.

These experiments lead to the conclusion, that the mechanical compression of the diaphragm and abdominal muscles had considerable influence in the production of vomiting; but it was conceived that this opinion would be confirmed, if, by removing the pressure from the stomach, vomiting did not take place. With this view the following experiment was made:

Four grains of emetic-tartar, dissolved in two ounces of water, was injected into the vein of a dog. (This mode of making the experiment is preferable to administering it by the stomach; for, by injection into a vein, vomiting is almost immediately excited; whilst, in the other way, it may not take place for an hour.) Immediately after the emetic had been injected, an incision was made through the abdominal muscles; and, immediately on the symptoms of vomiting taking place, the whole of the stomach was drawn through the opening, which did not prevent the efforts to vomiting taking place; for the animal made the same exertions to vomit, but without any effect, the stomach remaining perfectly quiescent. The effect of pressure upon that viscus was then tried, by placing the right hand upon the anterior portion, and the left on the posterior. Scarcely was the pressure commenced, than the efforts to vomit took place; that is, forcible contractions of the diaphragm and abdominal muscles occurred: on the pressure being suspended, the contraction of the diaphragm and abdominal muscles ceased immediately; and, on repeating the pressure, the muscular contractions were again renewed; and this was repeated six or seven times in succession, so as to leave no doubt of the connection subsisting between the pressure and the contraction of the abdominal muscles and the diaphragm. On the pressure being made with but a slight degree of force, then vomiting immediately took place, even without the previous administration of an emetic.

The next question requiring to be elucidated was, how far the contractions of the diaphragm and abdominal muscles might be independent of the action of the emetic on the stomach; and how far, instead of having an especial action on that viscus, may it produce a direct influence upon the above muscles. The following experiment was made to determine this question:

An opening was made into the abdomen of a dog, and the stomach was then removed; the vessels being afterwards secured, (a dog will survive this operation forty-eight hours,) the wound was then closed by sutures; afterwards two grains of emetic-tartar were dissolved in an ounce of water, and injected into the crural vein. Scarcely was the injection finished before nausea commenced, and the efforts to vomit seemed even more violent than under ordinary vomiting. After a quarter of an hour the dog seemed tranquil. The injection was again repeated in the same quantity, and with similar results; and the experiment was made six times, and constantly occasioned the same effects.

The last experiment I shall notice was made on a middle-sized dog. The stomach was removed, as in the former experiment, and a bladder substituted in its place, which was fixed to the œsophagus of the dog by means of an elastic gum catheter. The bladder contained half a pound of water, which distended but did not entirely fill it. The abdominal wound was then closed by means of sutures; four grains of tartar-emetic were then injected into the jugular vein; nausea immediately occurred, which was soon followed by efforts to vomit, which was finally accomplished, and the water was evacuated from the bladder.

These experiments of Magendie sufficiently point out the importance of the action of the muscles in the operation of vomiting, and this can only be explained by the agency of the brain and nervous system; but I think he has pushed this theory too far: for there is a great difference between a bladder with no obstacle to overcome at its orifice, and the stomach with its firmly-contracted cardia.

A certain regular action of the stomach itself is necessary, in order to effect the dilatations of the cardiac orifice; and, without this dilation, vomiting is never produced.

The necessity of this connection of the stomach and voluntary muscles with the brain, will explain why vomiting is excited with so much difficulty in cases where there is compression or other disturbance of the functions of the brain.

Four or five times the quantity of tartar-emetic, or even more, will be required to excite vomiting, because the brain cannot be roused by the stomach to produce the necessary accompanying actions of the diaphragm and abdominal muscles; and it is by no means an uncommon practice, on such occasions, to resort to violent medicines, such as large doses of white or blue vitriol. This is very improper practice; for the stomach in these cases may retain all its natural sensibility, and inflammation of it will be produced by such medicines, without expressions of pain warning us of the event.

An instance has occurred to my knowledge of a fatal inflammation of the stomach having been thus produced. The example was in a man subject to violent attacks of epilepsy of long duration; the cause of which was discovered, after death, to have been induced by a large tumor growing from the interior of the cranium. The medical practitioner, who had long attended him, found by experience that, when he could produce vomiting, the convulsions disappeared and sensibility returned. The prescription he administered consisted of a scruple of sulphate of zinc and ten grains of tartar-emetic dissolved in four ounces of water; of which mixture a table-spoonful was introduced into the stomach every quarter of an hour. It however became gradually more and more difficult to excite vomiting; and the successor of this practitioner increased the dose without restraint, on each recurrence of the disorder. On the last occasion, one drachm of sulphate of zinc and ten grains of tartar-emetic were administered within two hours, but no vomiting was produced. The patient however recovered from his epileptic paroxysm, but died a few days afterwards: and dissection showed that death had been occasioned by the injudicious administration of the medicine; for the stomach exhibited evident marks of severe inflammation.

What I have stated with respect to injuries of the head, apoplexy, and epilepsy, we find illustrated in cases of drunkenness, so long as the intoxicating fluids received into the stomach only disturb the functions of the brain: for example, before the coming-on of the insensible and subapoplectic state, and on its going off, there is nausea and vomiting; but, so long as the body remains in the state strongly resembling apoplexy, then the functions of the stomach remain quiescent. To give strong emetics in such a state would be highly improper; the stomach being unable to obtain the aid of the nervous influence of the brain, vomiting cannot be elicited; and, consequently, a local inflammation may be induced by such treatment.

The same want of association of action between the abdominal viscera and the voluntary muscles, will also show why retention of the urine and feces should so commonly take place in apoplexy, and all other cases of compression, or great depression of the functions, of the brain. I have known two cases in which the bowels resisted all the efforts of opening medicines; the cause of which was found on dissection, in both instances, to have arisen from abscesses formed in the brain, though there had never been any symptoms in either case to lead the practitioner to suspect such an event.

In some paralytic cases, signs of disorder of the stomach and intestines may be observed, such as indigestion, furred tongue, constipated bowels, and dark-coloured stools.

But it is not in instances of injuries of the head, apoplexy, or paralysis, that we find a difficulty to determine the nature of the disorder, or that a rational practitioner can be at a loss with respect to the treatment he ought to pursue. It is in ascertaining the seat of disease in children that we find so much difficulty.

By taking a cursory view of the condition of the brain, nervous system, and mucous membranes, during early life, we shall be enabled, I think, to point out the cause of this obscurity.

The brain in the fetus is of considerable size; and, long after birth, and nearly through the whole period of growth, the nervous system, and its centre the brain, predominate by their development over the other systems. Still this predominance is not uniformly the same at all the different stages. It goes on diminishing until puberty, at which time the nervous system is in equilibrium with the others; and at that epoch the genital organs succeed in the superiority.

This predominance of the nervous system in infancy influences on the one hand the sensations, and on the other the voluntary motions.

The first influence is very strongly marked. Infancy is the period of sensations: as every thing is then new, every object attracts the attention, and calls forth the powers of the senses. That which to us is indifferent, to it is a source of pleasure. It was therefore necessary that the cerebral nervous system should be accommodated, by its early development, to the great activity which is then required.

In fact, all the organs which receive external impressions, the nerves which transmit, and the brain which perceives them, are, during the time the infant is awake, in constant action. This must fatigue him much more than the adult, to whom, from repetition, these external objects become indifferent. So we remark that the period of activity is far shorter in infancy; the organs are wearied in a few hours, and consequently the necessity for sleeping returns more frequently; and in them that state of intermittance of action is more profound. It rarely happens that children, for the first few months, can remain the entire day awake, particularly if they have witnessed a great variety of objects. You may keep them longer awake, by withdrawing them from the light, sound, &c.

The multiplicity and frequency of sensations in the infant necessarily lead him to a number of motions, which have no force on account of the weakness of the muscles, but which, like the sensations, are very numerous. The eyes present new objects momentarily before him; he perpetually, therefore, wishes to touch them. It is then necessary that the nerves which serve to transmit the principle should be accommodated by their development, like those of the sensations to the continual action in which they are placed.

These two circumstances, the great development of the nervous system, and frequency of its action, in the infant, occasion its disorders to be those which predominate at that age.

Such is then the susceptibility of the brain to answer the sympathetic excitations, that a slight pain in any part determines immediately convulsions, which are, consequently, so much more frequent at that age than in the others. Thus worms, teething, or any other slight irritation, in a child, will occasion convulsions, which at a more advanced period of life would induce little or no effect.

We may remark, on this subject, that the different systems are more or less disposed in the different ages to answer to the sympathies, according as their predominance in the economy is more or less marked.

The same morbific cause fixed in any given organ, and which occasions convulsions in the infant, by acting sympathetically upon its brain, will, in a young girl, occasion suppression of the menses, by influencing the uterus, which then begins to predominate. In a strong young man, they may occasion a peripneumony. In an adult, where the gastric viscera predominate, an attack of some disease of those viscera, &c. Thus it is that the same passions which occasion at one period of life icterus, or inflammation of the liver, &c. more particularly induce in infancy epilepsy, or other convulsive affections.

Not only the nervous functions are frequently altered by sympathy in the infant, but it is more particularly at that age that we find the most numerous examples of organic injuries of the brain, medulla spinalis, nerves, or organs dependant upon them; of which, the fungus cerebri, hydrocephalus, spina bifida, are evident proofs. The great quantity of blood which then arrives at the nervous system has a great influence upon that system, and that quantity itself is called for by the predominance of the vital powers.

In proportion as the infant advances in size, its nervous system and the brain, which is the centre, gradually lose their characteristic predominance. Their diseases become less frequent, and they at last arrive on a level with the other systems.

These observations are more particularly applicable to the uncultivated state of society; for education frequently maintains in action, during the greater part of life, the predominance of the cerebral functions; and from this cause a morbid sensibility of the brain and nervous system is frequently developed, which serves to perpetuate acquired diseases and generate new ones. Hence dyspepsia, which in the savage or ignorant peasant is merely a disease of the gastric organs, in other states of cultivated society frequently induces severe disorder of the brain; this viscus being in such a state of sensibility as to become affected by sympathizing with the diseased stomach. I therefore consider hypochondriasis as a symptom of dyspepsia, existing only in persons whose brain and nervous system is in a state of morbid sensibility. I say morbid sensibility, because we do not find those individuals who are endowed with the highest range of intelligence and refined judgment, immediately on becoming dyspeptic, consider themselves as afflicted with every disease to which the human frame is incident: we do not find such persons constantly wasting their time in the perusal of medical books, consulting quacks, and hunting after specifics.

To render this subject more perspicuous, and show why the sympathies between the brain and alimentary canal should be more intimate in the infant than in the adult, we must also take a cursory view of the condition of the mucous membranes during the early periods of life; and then we shall be no longer at a loss to discover why there is so much difficulty in determining whether many of the diseases of children are those of the alimentary canal or of the brain.

The mucous secretions in the fetus are nearly quiescent; but, after birth, respiration and digestion commence. The mucous system, strongly excited by the numerous new agents with which it comes in contact, such as air, food, urine, bile, &c. acquires increased energies, and hence becomes more sensible of impressions.

The mucous membranes of the alimentary canal during early life retains its softness, or pulpy state; and this, probably, is one of the reasons why, during the period of lactation, they are unable to accommodate themselves to solid aliments, on account of the irritation they excite; and also why, until puberty, when they undergo marked changes, become thicker, firmer, and less vascular: I say, until these changes take place, we find stimulants frequently induce disorders of this organ.

The mucous fluids abound during early life: the pituitary membrane is then more moist. The bronchiæ are also frequently in a diseased state. The stomach and intestines are then also subject to a sort of catarrh, occasioning those diarrhœas with which we have frequently to contend. From the greater activity of the circulation in the mucous membranes at that time, they are more liable to spontaneous hæmorrhages: hence the frequency of epistaxis, hæmoptysis, &c.

Will not this also enable us to explain why individuals are more susceptible during infancy to scarlatina, measles, small-pox, &c. than in more advanced periods of life? and will not the same reasoning aid us also in accounting for the more frequent appearance of hooping-cough and croup at the same epoch.

Are we not therefore justified in concluding, from the physiological views we have just taken, that the great development and constant excessive action of the brain and nervous system in infancy is the cause of the prevalence of hydrocephalus, convulsions, and other nervous disorders, during infancy; and also that the high degree of vascularity and soft delicate structure of the mucous linings of the alimentary canal and bronchiæ, render such parts more susceptible of diseases in general in children than in adults. From these conditions of the brain and mucous membranes, we may easily conceive why diseases of the one system should extend their baneful influence to the other; and also why, when these organs arrive at an equilibrium with the rest of the body, and their sensibility becomes less acute by habit in the adult, such sympathies should be less powerfully developed.

*Hydrocephalus.*—Among the prominent symptoms of this disorder we must remark, severe pain of the head, delirium, dilatation of the pupils of the eye, and strabismus. But, besides these, there are numerous symptoms of a diseased condition of the stomach and intestines; and, even in idiopathic hydrocephalus, during the first stages, there is want of appetite, frequent vomiting, and the tongue is covered with a white crust. There is also great thirst. The bowels are generally constipated; the alvine evacuations are green or black, and sometimes intolerably fetid.

In the early stages of hydrocephalus, it is difficult to distinguish it from worms in the alimentary canal, because the same symptoms occur in both disorders. Both commence with slow fever. In both there is loss of appetite, vomiting, pain of the head, delirium, and convulsions. We are therefore frequently at a loss to determine the precise nature of the disorder until it has arrived at its second stage.

The most experienced practitioners, and amongst others we must not omit the respectable name of Fothergill, have declared it almost impossible to form a just diagnosis.

How often do we find, on dissection, that a disease, which we had taken for hydrocephalus, has been only a disorder of the alimentary canal; the sympathetic symptoms predominating over those of the real disorder. And again, how frequently does it happen, that irritation of the brain, first occurring from sympathy with the alimentary canal, finishes at last by becoming the more prominent disorder.

We have frequently been guided to a correct diagnosis by observing the period at which vomiting takes place. When the disease is of the brain, or hydrocephalus, the vomiting will occur immediately on waking, or on the head being agitated. But when the disorder is of the alimentary canal, the vomiting will more particularly take place after taking food.

*The Febris Infantum Remittens.*—This assuredly is a disorder of the alimentary canal, and the fever is merely symptomatic of the derangement of the intestines; yet it does not a little resemble hydrocephalus internus. It is not my intention at present to attempt a diagnosis between them. It is sufficient for me to observe, that, from the brain and stomach being considerably disordered in both of them, it is frequently difficult to form a just determination which is the primary affection.

*Tabes Mesenterica*, or hectic from disease of the mesenteric glands, is a very frequent disease. It is not often met with before the time of weaning, nor after puberty, and but seldom after the age of eight or ten years.

This disease I have been able to trace, from numerous dissections, to irritation or disorganization of the mucous membranes of the alimentary canal. The glands in the neighbourhood of the diseased intestine first take on the inflammatory action, and I have frequently been able to find spots of ulceration in the mucous membrane; from which I have seen inflamed lymphatics and turgid blood-vessels radiating to the neighbouring glands, which have been enlarged in proportion to their contiguity to the diseased intestines.

This disorder is evidently induced by improper food received into the alimentary canal, before the organization of the mucous membrane has acquired that degree of firmness which can enable it to resist their baneful influence. From the appearances found on dissection we may readily account for the irregular state of the bowels, being at one time costive, and at another passing dark loose feces.

The disease being chiefly confined to the small intestines, more particularly the ileum, the functions of the stomach are not much disturbed, and we do not find such powerful sympathetic affection of the brain as when the stomach and commencement of the duodenum are the seat of disorder.

The appetite being good, and digestion going on well, at the same time that the belly is hard and somewhat tumid, whilst the body gradually wastes away, are sufficient characteristics of tabes mesenterica.

It is foreign to my present object to enter into the other symptoms of the disease.

*Epilepsy of young persons*, is very frequently occasioned by irritation of the alimentary canal, from various causes, but especially from worms.

The examples we have here given may suffice to illustrate the proposition, that the state of organization of the brain and mucous membranes of the alimentary canal, in infancy, renders them peculiarly liable to re-act on each other, and often occasions much difficulty in forming the diagnosis of their respective diseases.

Our time will not permit us to attempt, at present, an elucidation of these interesting questions. But I may be allowed to remark, that, if the delicacy of the organization of the mucous membranes will not enable them to support the presence of those aliments which, at a more advanced state of life, are the most proper for the nutrition of the adult, how can we expect that it will be equal to struggle against the drastic purges and powerful emetics so liberally employed by the routine practitioners of the present day.

Before I take leave of this subject, I should solicit attention to a fact which has long interested the public. Habitual drunkards have been known, in all times, to have not unfrequently attained old age; and various have been the explanations given of this circumstance. The views we have just exposed will enable us to explain this apparent incongruity with more ordinary results. Such individuals will be found, on investigation, to have generally contracted the pernicious practice of drinking, after the mucous membranes of the alimentary canal had lost the predominance they possessed in early life. But, whenever the habit of drinking ardent spirits had been commenced and persevered in before that time, there have been but few examples recorded of such persons attaining longevity.

In the army, where there are the best means of putting this statement to the test of experience, I have uniformly found, on enquiry, that the oldest officers had been temperate in their youth, or became soldiers after they had arrived at manhood. Spirits are as destructive to the alimentary canal before maturity, as animal food during the period of lactation.*

[The first lecture was on the Anatomy and Physiology of the Skin and Mucous Membranes, showing their similarity in respect to structure and functions. The anatomical museum recently presented by Dr. Bailey to the College of Physicians, furnished ample means of illustrating these points. This lecture will be given in some future Number.]

---

* The following account, taken from a "*Birmingham Chronicle*" of last month, may be brought forward, with a multitude of other similar examples, in confirmation of this proposition.

"On Monday, the 26th ult. an inquest was held, before John Tomes, Esq. on the body of a child, four years old, who had swallowed a quantity of gin. After deliberating four hours, the jury returned a verdict of 'Died in consequence of excessive drinking.' A man who lodged with the parents of the deceased had been drinking at the White Lion, Warwick: the child being sent to fetch him home, he ordered a glass of gin for it, which the child drank; and, being asked if he should like another, he replied in the affirmative. Another glass was accordingly procured, and drank by the deceased; the landlord remarking at the time that he thought it would be too much for him. The child walked home; but in a few hours after was seized with convulsions, and continued in that state until four o'clock next morning, when he expired."

